# Work stress and changes in heart rate variability among employees after first acute coronary syndrome: a hospital-based longitudinal cohort study

**DOI:** 10.3389/fpubh.2024.1336065

**Published:** 2024-03-26

**Authors:** Zhao Hu, Xingyu Cao, Pan Jing, Bangying Zhang, Yunke Shi, Johannes Siegrist, Jian Li, Min Zhang

**Affiliations:** ^1^Cardiology Department, The First Affiliated Hospital of Kunming Medical University, Kunming, China; ^2^Institute of Medical Sociology, Centre for Health and Society, Faculty of Medicine, Heinrich Heine University Düsseldorf, Düsseldorf, Germany; ^3^Departments of Environmental Health Sciences and Epidemiology, Fielding School of Public Health, School of Nursing, University of California, Los Angeles, Los Angeles, CA, United States

**Keywords:** acute coronary syndrome, effort-reward imbalance, heart rate variability, job strain, work stress

## Abstract

**Background:**

Work stress is considered as a risk factor for coronary heart disease, but its link with heart rate variability (HRV) among heart attack survivors is unknown yet. The aim of this study was to investigate associations between baseline work stress and the changes of HRV over one-year after onset of acute coronary syndrome (ACS).

**Methods:**

Hundred and twenty-two patients with regular paid work before their first ACS episode were recruited into this hospital-based longitudinal cohort study. During hospitalization (baseline), all patients underwent assessments of work stress by job strain (JS) and effort-reward imbalance (ERI) models, and were assigned into low or high groups; simultaneously, sociodemographic and clinical data, as well depression, anxiety, and job burnout, were collected. Patients were followed up 1, 6, and 12 months after discharge, with HRV measurements at baseline and each follow-up point. Generalized estimating equations were used to analyze the effects of baseline work stress on HRV over the following 1 year.

**Results:**

After adjusting for baseline characteristics and clinical data, anxiety, depression, and burnout scores, high JS was not associated with any HRV measures during follow-up (all *p* > 0.10), whereas high ERI was significantly related to slower recovery of 5 frequency domain HRV measures (TP, HF, LF, VLF, and ULF) (all *p* < 0.001), and marginally associated with one time domain measure (SDNN) (*p* = 0.069). When mutually adjusting for both work stress models, results of ERI remained nearly unchanged.

**Conclusion:**

Work stress in terms of ERI predicted lower HRV during the one-year period after ACS, especially frequency domain measures.

## 1 Introduction

Work stress has been identified as a risk factor for coronary heart disease (CHD). Several studies have revealed that work stress was associated with an increased risk of CHD by 20% (1–3), where work stress was widely defined by two theoretical models, the job strain (JS) model and the effort-reward imbalance (ERI) model. The former model focuses on task characteristics of combination of high job demand and low job control (4); while nonreciprocal social exchange, i.e., high cost and low gain at work, is emphasized in the latter model (5). Though several biological pathways have been proposed to explain the mechanisms between work stress and CHD (6, 7), heart rate variability (HRV) has received special attention given its powerful clinical prediction to heart attack (8). HRV refers to the variation in time interval between successive heartbeats, reflecting the balance between sympathetic and parasympathetic activity which is a measure of the autonomic nervous system’s influence on the heart (9). Recent studies have revealed that a decrease in HRV or a blunted increase in HRV is associated with both acute and chronic stress, suggesting that HRV can function as a marker of stress (10, 11). In the field of research on work stress and HRV, studies have shown that work stress in terms of both JS and ERI was associated with lower levels of HRV among apparently healthy workers (12, 13).

Clinical management of CHD has experienced significant progress over the past two decades, leading to substantial improvement in patient survival rates post-CHD (14). Despite these advances, approximately one-third of CHD patients still face a considerable risk, estimated to be between 20 and 30%, of experiencing recurrent cardiovascular events during the subsequent 10 years (15). Interestingly, a handful epidemiological studies indicated that, among workers with CHD, the risk of subsequent cardiac events was increased by 65% when exposed to JS or ERI, as suggested by our previous meta-analysis (16, 17). Regarded as an important prognostic marker in various cardiovascular diseases (CVD), HRV can predict increased risk of adverse outcomes (18). Especially in patients after myocardial infarction (MI), decreased HRV has been associated with an increased risk of arrhythmia events and elevated mortality rate (18, 19), suggesting that monitoring HRV may help identify those at greater risk of future cardiac events following MI. Hypothetically, HRV might also play an important role in explaining the bio-medical pathway from work stress to recurrent cardiac events. Yet, to the best of our knowledge, the link between work stress and HRV among employees who survived from heart attack was not explored.

Therefore, with an attempt to provide scientific evidence by exploring the potential role of HRV in explaining work stress and recurrent CHD, we carried out a longitudinal study among patients with first acute coronary syndrome (ACS), which is a severe condition of CHD, and aimed to investigate associations of work stress, being measured by both JS and ERI, with HRV over a 12-month course of post-ACS period. In addition, we compared the explanatory power of JS and ERI to predict changes in HRV.

## 2 Methods

### 2.1 Study subjects

**Figure 1:**
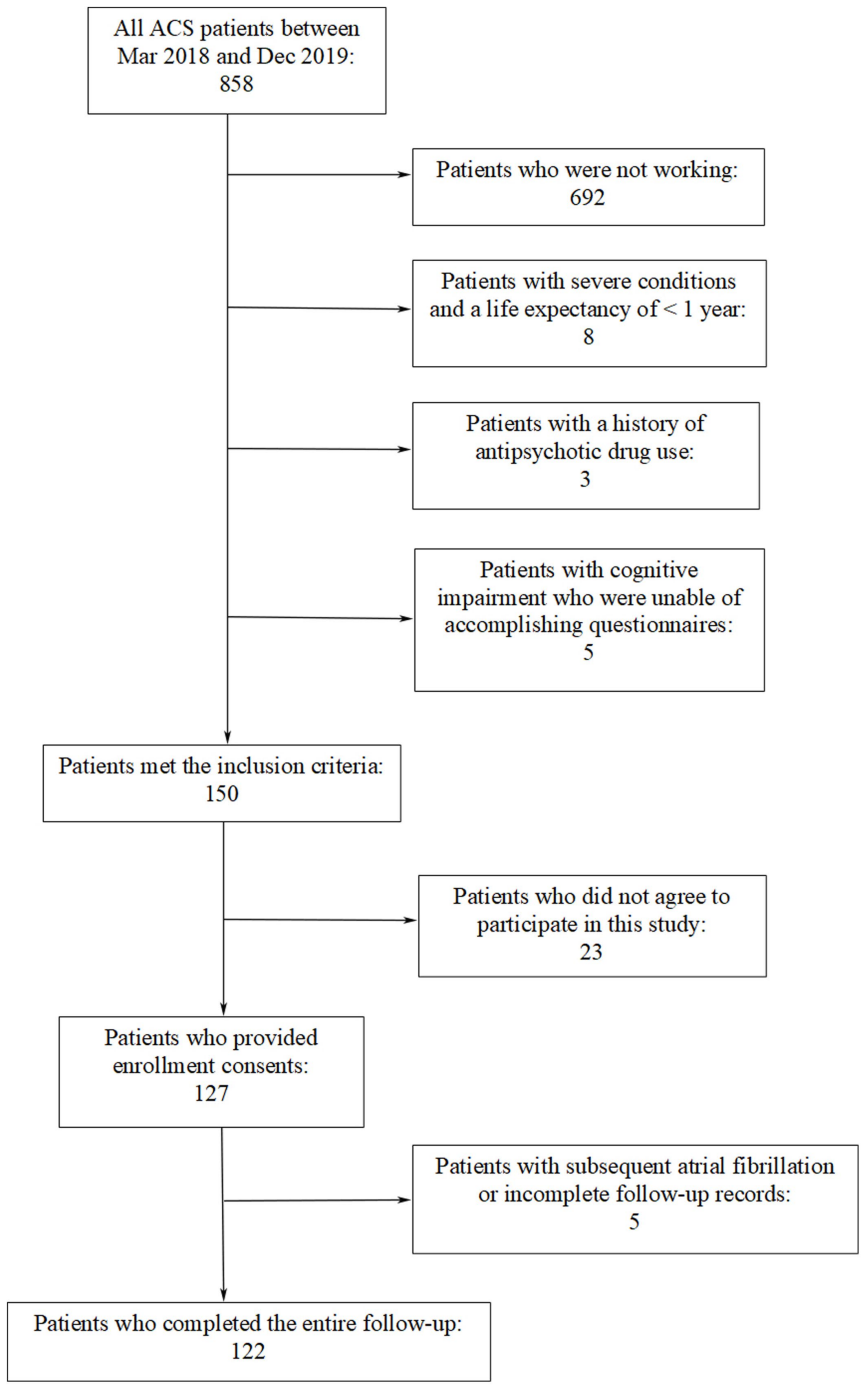
Study subjects recruitment.

Patients admitted to the First Affiliated Hospital of Kunming Medical University between March 2018 and December 2019 participated in this study. They had experienced their first ACS episode and previously maintained regular paid employment prior to the ACS event. ACS covers various acute myocardial ischemic states including ST-segment elevation myocardial infarction (STEMI), non-ST segment elevation myocardial infarction (NSTEMI) and unstable angina (UA) (20, 21). Exclusion criteria included arrhythmia frequency disrupting the investigation of HRV, potential comorbidities influencing symptom onset, and severe systemic disease. Patients with a history of antipsychotic drug use, those were incapable of accomplishing questionnaires even with assistance, and individuals who refused or were unable to undergo a 24-h Holter ECG monitoring were also excluded from the study. Coronary angiography was performed for every included patient during the hospital stay period. Figure 1 is a flowchart showing that 122 patients were ultimately recruited into this study.

### 2.2 Study design

In this hospital-based longitudinal cohort study, patients’ work stress levels were evaluated during their hospital stay, which served as the study baseline. Participants were subsequently designated into either low or high work stress groups (see below for details). Additionally, sociodemographic and clinical data, as well as anxiety, depression, and job burnout levels, were collected at baseline. Follow-ups were conducted 1, 6, and 12 months post-discharge, with HRV parameters measured at baseline and at each follow-up for a total of four times. The Ethics Committee of Kunming Medical University (Kunming, China) issued an approval for the study, which was carried out in accordance with the Declaration of Helsinki guidelines. The informed consent form was signed by all subjects.

### 2.3 Demographic and medical data collection

Clinical and demographic data were extracted from patients’ medical records. This information included age, sex, education level, number of family members, monthly family income, type of ACS experienced (UA, STEMI, NSTEMI), Killip’s grade at admission, prior medical history such as hypertension, diabetes, hyperlipemia, and stroke, current health-related behaviors including smoking and heavy drinking, along with family medical history with regards to CVD and medication usage.

### 2.4 Work stress evaluation

The Job Content Questionnaire (JCQ) and the ERI Questionnaire corresponding to the JS and ERI models, respectively, were used to evaluate work stress. The JCQ, developed by Karasek (4), has been demonstrated to be reliable and valid in its Chinese version (22). For the present study, a shortened version of the JCQ was utilized, highlighting job demand and control with four questions (two items for demand and two items for control), which was highly consistent with the original (23). The ERI Questionnaire, developed by Siegrist (5), also has a reliable and valid Chinese version (24). In this study, we used a simplified ERI Questionnaire consisting of nine questions concerning effort and reward (three items for effort and six items for reward), which was in parallel with the original version (25). Then, two groups were dichotomized based on the JS and ERI results, i.e., low (ratio ≤ 1) and high (ratio > 1). In the present study, Cronbach’s α coefficients were 0.77 for job demand, 0.75 for job control, 0.77 for effort, and 0.79 for reward.

### 2.5 Anxiety, depression, and job burnout assessment

As previously recognized, anxiety and depression are important risk factors that impact CHD prognosis (26, 27). Job burnout is another significant factor associated with poor prognosis in ACS according to our past study (28–30). These three factors were assessed before discharge as confounding factors in this study. Anxiety and depression were evaluated with the 14-item Hospital Anxiety and Depression Scale (HADS) with HADS-anxiety and HADS-depression subscales. Both subscales were scored 0–21 points, where a higher score suggested more severe anxiety and depression symptoms (31). This scale has successfully applied in Chinese CHD patients (32). The total HADS score, which is the sum of the anxiety and depression scores (33), was used to adjust for the impact of anxiety and depression on HRV in subsequent statistical analysis. Job burnout was measured with the job burnout subscale of the Copenhagen Burnout Inventory (CBI). With a range of 0–100 points, a high CBI score represented more severe job burnout (34). The Chinese version of CBI has been shown to be reliable and valid by other studies (35). In the current study, Cronbach’s alpha coefficients were 0.71 for HADS-anxiety, 0.73 for HADS-depression, and 0.72 for the CBI job burnout subscale, respectively.

### 2.6 HRV measurement

HRV was measured at four time points: 1 day prior to discharge, and 1, 6, and 12 months post-discharge. Eligible subjects underwent 24-h Holter monitoring between 08:00 and 09:00 AM, with continuous electrocardiogram (ECG) signals recorded using a 12-channel Holter system (Biomedical Systems, USA). The hardware of the system sampled at a rate of 500 Hz. The recorded analog ECG signals were digitized and analyzed using the BMS Century 3,000 software package for HRV analysis (version 2.0). Before calculating the HRV parameters, the raw RR interval data were processed to remove artifacts. Two specific criteria were applied to identify artifacts within the RR interval data: An RR interval was flagged as an artifact if it was outside the 300–2000 ms range, or if the ratio of a consecutive RR interval to its predecessor was not within the 0.8–1.2 range. If artifacts represented less than 5% of the total RR intervals, and there were no extended sequences of consecutive artifacts, the HRV recording was deemed to meet quality standards; otherwise, a new recording was required. For HRV recordings that passed the quality assessment, artifacts were removed, and linear interpolation was employed to estimate the values of missing RR intervals (9). HRV parameters were calculated with ECG data over the entire 24-h period. The standard deviation for all normal-to-normal RR intervals (SDNN) and the root mean square of successive differences (RMSSD) were derived as the common time-domain parameters for HRV. Fast Fourier transform was utilized for transformation of the heart rate power spectrum from a 24-h normal RR interval, yielding five frequency-domain parameters covering high frequency power (HF = 0.15–0.40 Hz), low frequency power (LF = 0.04–0.15 Hz), very low frequency power (VLF = 0.0033–0.04 Hz), ultra low frequency power (ULF < 0.0033 Hz), and total power (TP ≤ 0.40 Hz). To accommodate the skewed distribution, the natural logarithms of seven HRV parameters obtained by conversion were as follows: ln(SDNN), ln(RMSSD), ln(TP), ln(HF), ln(LF), ln(VLF), and ln(ULF), which were in line with normal distribution. All HRV data collection and analysis were performed according to the guidelines for reporting articles on psychiatry and heart rate variability (GRAPH) (36).

### 2.7 Statistical analysis

All data were processed and analyzed using Stata 10 (Stata, College Station, TX, USA). Mean ± standard deviation (SD), number and percentage (%) were used to express continuous and categorical variables, respectively. First, the t-test or χ2 test was conducted to compare baseline characteristics between high and low work stress groups. Second, repeated measures analysis of variance (ANOVA) was applied to study the variations of HRV measures over a 12-month period. Third, generalized estimating equations (GEEs), a statistical method for dealing with repeated measurement data (37, 38), were employed to examine the longitudinal associations of JS or ERI models at the onset with shifts in HRV measures at different intervals (baseline, together with 1, 6, and 12 months). GEE regression was modeled via four steps. Demographic and clinical variables from the baseline were incorporated into Model I. The HADS sum score was also included in Model II, which accounts for depression and anxiety. Model III built upon Model II with further adjustment for job burnout. Notably, we looked at the effects of JS or ERI on HRV individually, without mutual adjustment from Model I to III. Ultimately, we estimated the link between JS, ERI, and HRV in Model IV by adjusting each work stress model for the others. Such mutual adjustment aimed to assess how each work stress model independently affected the longitudinal changes in HRV (39).

## 3 Results

### 3.1 Characteristics of subjects at baseline

As is indicated in Figure 1, a total of 122 patients (aged between 27 and 62 years with a median age of 49.5 years; 103 males and 19 females) participated in the study. No cardiac events such as cardiac death, heart failure, recurrent ACS events, and rehospitalization due to ACS-related symptoms were observed during the follow-up period. The characteristics of subjects at baseline are presented in Table 1. There were significant differences in job burnout score and HADS score between the high JS group and the low JS group. Between the high ERI group and the low ERI group, there were statistical differences in ACS type and job burnout score. Other characteristics did not show significant differences between the high/low JS groups, or the high/low ERI groups.

**Table 1:** Characteristics of subjects at baseline (*n* = 122).

	Total	JS			ERI		
		Low work stress (*n* = 56)	High work stress (*n* = 66)	*p*	Low work stress (*n* = 53)	High work stress (*n* = 69)	*p*
Age (y)	49.49 ± 7.49	48.84 ± 8.27	50.05 ± 6.78	0.378	48.11 ± 7.92	50.55 ± 7.02	0.075
Male [*n*(%)]	103 (84%)	47 (84%)	56 (85%)	0.889	46 (87%)	57 (83%)	0.528
Education level [*n*(%)]							
Primary school or below	34 (28%)	14 (25%)	20 (30%)	0.867	10 (19%)	24 (35%)	0.151
Junior high school	47 (39%)	22 (39%)	25 (38%)		22 (41%)	25 (36%)	
High school	16 (13%)	7 (13%)	9 (14%)		10 (19%)	6 (9%)	
Junior college/College or higher	25 (20%)	13 (23%)	12 (18%)		11 (21%)	14 (20%)	
Number of family members (*n*)	3.99 ± 1.31	3.88 ± 1.25	4.09 ± 1.36	0.366	3.89 ± 1.15	4.07 ± 1.42	0.439
Monthly family income (THOUS Yuan)	7.28 ± 2.14	7.19 ± 1.70	7.35 ± 2.46	0.671	7.41 ± 1.68	7.17 ± 2.44	0.552
ACS type [*n*(%)]							
UA	18 (15%)	9 (16%)	9 (14%)	0.927	12 (23%)	6 (9%)	0.020
STEMI	57 (47%)	26 (46%)	31 (47%)		18 (34%)	39 (56%)	
NSTEMI	47 (38%)	21 (38%)	26 (39%)		23 (43%)	24 (35%)	
Killip’s grade at admission [*n*(%)]							
Grade I	54 (44%)	24 (43%)	30 (45%)	0.935	23 (43%)	34 (49%)	0.872
Grade II	49 (40%)	22 (39%)	27 (41%)		22 (42%)	26 (38%)	
Grade III	15 (13%)	8 (14%)	7 (11%)		7 (13%)	7 (10%)	
Grade IV	4 (3%)	2 (4%)	2 (3%)		1 (2%)	2 (3%)	
Medical history [*n*(%)]							
Hypertension	52 (43%)	25 (45%)	27 (41%)	0.678	18 (34%)	34 (49%)	0.090
Diabetes	29 (24%)	12 (21%)	17 (26%)	0.576	15 (28%)	14 (20%)	0.303
Dyslipidemia	44 (36%)	23 (41%)	21 (32%)	0.289	23 (43%)	21 (30%)	0.139
Stroke	6 (5%)	3 (5%)	3 (5%)	0.836	1 (2%)	5 (7%)	0.175
Family history of CVD	39 (32%)	21 (38%)	18 (27%)	0.227	21 (40%)	18 (26%)	0.112
Smoking at present	83 (68%)	42 (75%)	41 (62%)	0.129	40 (75%)	43 (62%)	0.123
Heavy drinker	40 (33%)	20 (36%)	20 (30%)	0.526	17 (32%)	23 (33%)	0.883
Medication [*n*(%)]							
Aspirin	122 (100%)	56 (100%)	66 (100%)	–	53 (100%)	69 (100%)	–
P2Y12 receptor antagonists	122 (100%)	56 (100%)	66 (100%)	–	53 (100%)	69 (100%)	–
Statin	122 (100%)	56 (100%)	66 (100%)	–	53 (100%)	69 (100%)	–
Beta blockers	122 (100%)	56 (100%)	66 (100%)	–	53 (100%)	69 (100%)	–
ACEI or ARB	122 (100%)	56 (100%)	66 (100%)	–	53 (100%)	69 (100%)	–
HADS score	13.56 ± 4.90	12.34 ± 4.42	14.59 ± 5.08	0.0108	12.85 ± 5.07	14.10 ± 4.73	0.162
Job burnout score	49.37 ± 17.07	43.44 ± 16.73	54.40 ± 15.80	0.0003	39.57 ± 15.25	56.90 ± 14.42	<0.001

Differences were determined by *t*-test for continuous variables, χ^2^ test for categorical variables. JS, job strain; ERI, effort-reward imbalance; ACS, acute coronary syndrome; UA, unstable angina; STEMI, ST-segment elevated myocardial infarction; NSTEMI, non-ST-segment elevated myocardial infarction; CVD, cardiovascular disease; ACEI, angiotensin-converting enzyme inhibitor; ARB, angiotensin receptor antagonist; HADS, Hospital Anxiety and Depression Scale. HADS score = anxiety score + depression score. JS-low work stress: demand/control ≤ 1; JS-high work stress: demand/control > 1. ERI-low work stress: effort/reward ≤ 1; ERI-high work stress: effort/reward > 1.

### 3.2 The HRV measures over 12 months after ACS

Table 2 presents the natural logarithmized HRV measures over 12 months after ACS. Two time domain measures (SDNN, RMSSD) and 5 frequency domain measures (LF, HF, VLF, ULF, and TP) gradually increased within 12 months, which was consistent with the variation pattern of HRV during ACS recovery process. The raw HRV measures can be seen in Supplementary Table S1.

**Table 2:** The natural logarithmized HRV measures over 12 months after ACS (mean ± SD).

HRV parameters	Baseline	1 Month	6 Months	12 Months	*p-*value ^a^
Time domain HRV					
Ln(SDNN) (ln ms)	4.92 ± 4.63	4.99 ± 4.52	5.13 ± 4.81	5.15 ± 4.39	0.0047
Ln(RMSSD) (ln ms)	4.26 ± 0.96	4.53 ± 0.85	4.58 ± 0.59	4.62 ± 0.85	0.1495
Frequency domain HRV					
Ln(TP) (ln ms^2^)	8.77 ± 1.89	9.31 ± 1.32	9.79 ± 1.16	10.33 ± 1.79	0.0086
Ln(HF) (ln ms^2^)	7.62 ± 1.50	7.68 ± 1.78	8.36 ± 1.76	8.89 ± 1.08	0.0326
Ln(LF) (ln ms^2^)	7.37 ± 1.32	7.55 ± 1.56	7.98 ± 1.51	8.20 ± 1.66	0.1351
Ln(VLF) (ln ms^2^)	7.58 ± 0.99	7.85 ± 1.05	8.29 ± 0.79	8.57 ± 0.99	0.0045
Ln(ULF) (ln ms^2^)	7.85 ± 0.98	8.05 ± 1.02	8.51 ± 1.12	9.36 ± 1.22	0.0021

SD, standard deviation; HRV, heart rate variability; SDNN, standard deviation of NN intervals; RMSSD, root mean square of successive differences; TP, total power; HF, high frequency; LF, low frequency; VLF, very low frequency; ULF, ultra-low frequency, Ln: natural logarithm. ^a^Repeated measures ANOVA. Time trends of HRV scores were examined by repeated measures ANOVA.

### 3.3 Associations of work stress at baseline with changes in HRV during 12 months after ACS

**Figure 2:**
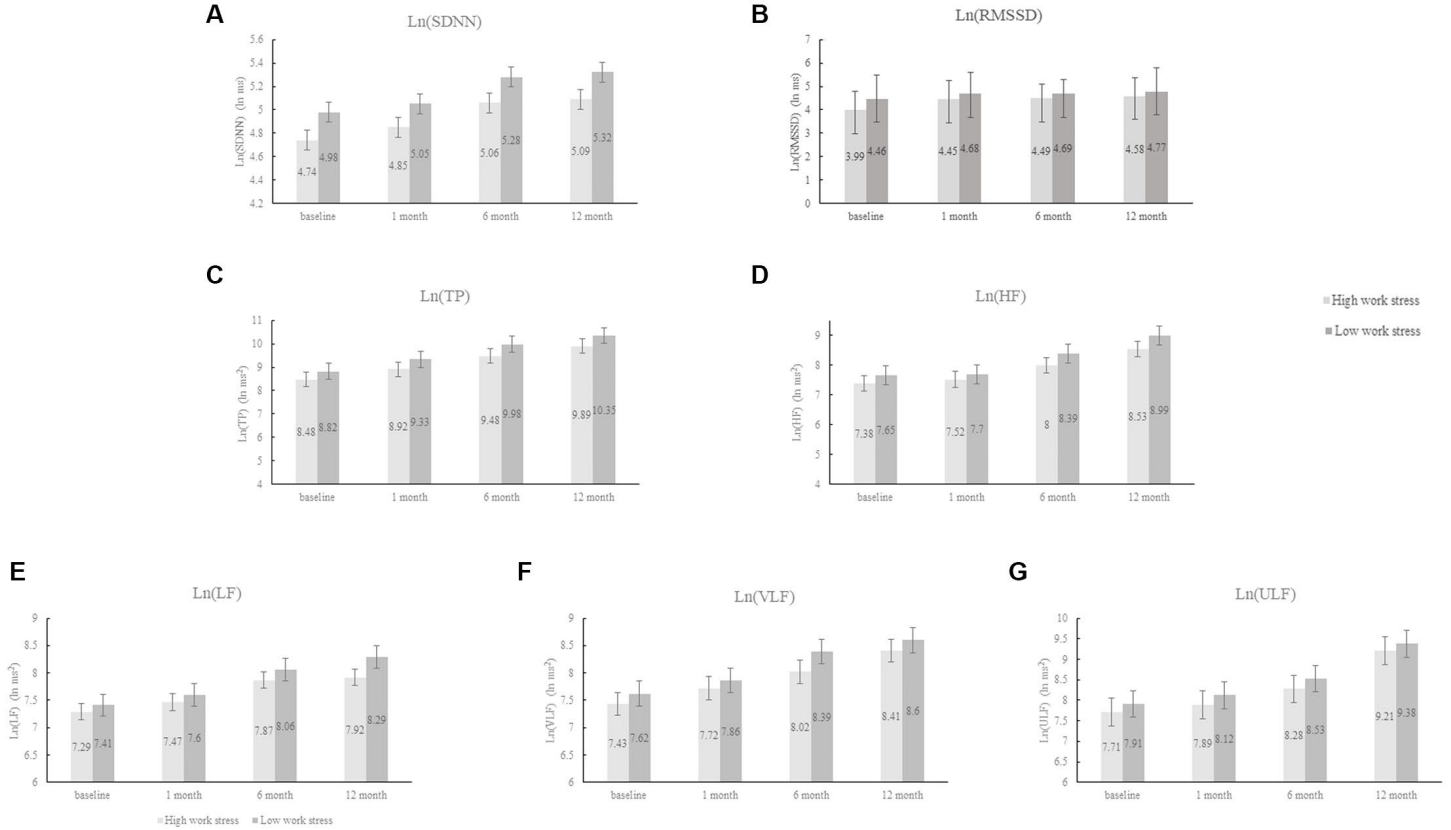
The changes in HRV between the ERI high and low groups. HRV, heart rate variability; ERI, effort-reward imbalance; SDNN, standard deviation of NNintervals; RMSSD, root mean square of successive differences; TP, total power; HF, high frequency; LF, low frequency; VLF, very low frequency; ULF, ultra-low frequency; Ln, natural logarithm.

After adjusting for demographic and clinical data, HADS score, and burnout score (Model III), work stress measured by JS was not associated with any HRV measures during the follow-up period (all *p* > 0.10); while high ERI was significantly related to slow recovery of 5 frequency measures of HRV (TP, HF, LF, VLF, and ULF) (all *p* < 0.001) and marginally associated with reduced one time domain of HRV (SDNN) (*p* = 0.069). The mutual adjustment for JS and ERI did not change the results (see Model IV). Figure 2 intuitively reflected the trend of HRV changes between the ERI high and low groups, which is consistent with Table 3.

**Table 3:** The coefficients and 95% CIs of repeated measures of HRV parameters during 1-year follow-up by work stress at baseline according to JS and ERI models.

Work stress		Model I		Model II		Model III		Model IV	
		Coefficient (95% CIs)	*p-*value	Coefficient (95% CIs)	*p-*value	Coefficient (95% CIs)	*p-*value	Coefficient (95% CIs)	*p-*value
		Ln(SDNN)							
JS	Low	0.00		0.00		0.00		0.00	
	High	−0.09 (−0.21, 0.04)	0.183	−0.06 (−0.19, 0.07)	0.381	−0.04 (−0.17, 0.09)	0.596	−0.01 (−0.14, 0.12)	0.913
ERI	Low	0.00		0.00		0.00		0.00	
	High	−0.20 (−0.33, −0.07)	0.003**	−0.18 (−0.32, −0.05)	0.007**	−0.14 (−0.28, 0.01)	0.069	−0.14 (−0.29, 0.01)	0.070
		Ln(RMSSD)							
JS	Low	0.00		0.00		0.00		0.00	
	High	−0.21 (−0.53, 0.11)	0.191	−0.23 (−0.57, 0.10)	0.171	−0.13 (−0.47, 0.21)	0.447	−0.10 (−0.44, 0.24)	0.554
ERI	Low	0.00		0.00		0.00		0.00	
	High	−0.35 (−0.63, −0.08)	0.011*	−0.37 (−0.64, −0.09)	0.010*	−0.27 (−0.62, 0.08)	0.133	−0.25 (−0.60, 0.10)	0.163
		Ln(TP)							
JS	Low	0.00		0.00		0.00		0.00	
	High	−0.52 (−0.94, −0.10)	0.016*	−0.34 (−0.76, 0.08)	0.115	−0.29 (−0.72, 0.13)	0.171	−0.06 (−0.46, 0.33)	0.752
ERI	Low	0.00		0.00		0.00		0.00	
	High	−1.23 (−1.63, −0.83)	<0.001***	−1.12 (−1.52, −0.72)	<0.001***	−1.04 (−1.48, −0.59)	<0.001***	−1.02 (−1.46, −0.58)	<0.001***
		Ln(HF)							
JS	Low	0.00		0.00		0.00		0.00	
	High	−0.57 (−1.10, −0.04)	0.036*	−0.36 (−0.88, 0.17)	0.182	−0.33 (−0.86, 0.20)	0.222	−0.08 (−0.59, 0.42)	0.749
ERI	Low	0.00		0.00		0.00		0.00	
	High	−1.25 (−1.78, −0.72)	<0.001***	−1.13 (−1.64, −0.62)	<0.001***	−1.02 (−1.59, −0.44)	<0.001**	−1.00 (−1.56, −0.44)	<0.001***
		Ln(LF)							
JS	Low	0.00		0.00		0.00		0.00	
	High	−0.30 (−0.74, 0.15)	0.192	−0.13 (−0.58, 0.31)	0.557	−0.09 (−0.53, 0.35)	0.691	0.12 (−0.30, 0.54)	0.562
ERI	Low	0.00		0.00		0.00		0.00	
	High	−1.09 (−1.52, −0.66)	<0.001***	−1.00 (−1.43, −0.58)	<0.001***	−0.93 (−1.40, −0.46)	<0.001***	−0.95 (−1.42, −0.48)	<0.001***
		Ln(VLF)							
JS	Low	0.00		0.00		0.00		0.00	
	High	−0.26 (−0.52, 0.01)	0.057	−0.17 (−0.44, 0.10)	0.214	−0.13 (−0.40, 0.14)	0.345	0.01 (−0.23, 0.25)	0.934
ERI	Low	0.00		0.00		0.00		0.00	
	High	−0.81 (−1.05, −0.57)	<0.001***	−0.77 (−1.01, −0.53)	<0.001***	−0.74 (−1.01, −0.47)	<0.001***	−0.74 (−1.01, −0.47)	<0.001***
		Ln(ULF)							
JS	Low	0.00		0.00		0.00		0.00	
	High	−0.26 (−0.56, 0.03)	0.082	−0.15 (−0.45, 0.14)	0.316	−0.13 (−0.43, 0.17)	0.409	0.05 (−0.22, 0.31)	0.723
ERI	Low	0.00		0.00		0.00		0.00	
	High	−0.96 (−1.23, −0.69)	<0.001***	−0.90 (−1.17, −0.64)	<0.001***	−0.91 (−1.21, −0.61)	<0.001***	−0.92 (−1.22, −0.63)	<0.001***

JS, job strain; ERI, effort-reward imbalance; CIs, Coefficient intervals; ACS, acute coronary syndrome; UA, unstable angina; STEMI, ST, segment elevated myocardial infarction; NSTEMI, non-ST-segment elevated myocardial infarction; CVD, cardiovascular disease; HADS, Hospital Anxiety and Depression Scale; HRV, heart rate variability; SDNN, standard deviation of NN intervals; RMSSD, root mean square of successive differences; TP, total power; HF, high frequency; LF, low frequency; VLF, very low frequency; ULF, ultra-low frequency; Ln, natural logarithm. Generalized estimating equations, *p < 0.05, **p < 0.01, ***p < 0.001. Model I: adjusted for age, sex, ACS type (UA, STEMI, NSTEMI), education level (primary school or below, junior high school, high school, junior college /College or higher), number of family members, monthly family income, medical history (hypertension, diabetes, dyslipidemia, stroke), family history of CVD, smoking at present, heavy drinker. Model II: Model I + additionally adjusted for HADS score. Model III: Model II + additionally adjusted for job burnout score. Model IV: Model III + JS and ERI were mutually adjusted for each other.

## 4 Discussion

This study provides new research evidence about a potential bio-medical pathway explaining the observed association of work stress with increased risk of recurrent CHD among employees (16, 17). An imbalance between high effort and low reward at work, assessed at baseline predicted lower HRV 1 year after ACS. For the alternative work stress model (i.e., JS) this association was not observed. To the best of our knowledge, this is the first study to reveal longitudinal associations between work stress and HRV changes in ACS patients, and it is also the first study to compare two work stress models in this regard.

HRV has been well recognized in determining prognosis of post myocardial infarction, and as an established clinical marker of sudden cardiac death. Back to 1987, a study showed that the mortality in post-MI patients with SDNN less than 50 ms was 5.3 times higher than those with SDNN more than 100 ms (40). And a meta-analysis in 2009 demonstrated that post-MI patients with SDNN below 70 ms had a 4-fold risk of death in the next 3 years (41). Regarding the frequency domain HRV measures, recent research suggested that decreased frequency power would elevate risk of adverse clinical outcomes among cardiac patients (42, 43). In particular, studies have shown that the reductions of VLF and ULF were associated with an increased risk of all-cause death and cardiac death (44–47). Our study revealed significant associations between higher work stress at baseline and slower recovery of HRV during one-year follow-up period. In this context, it seems plausible to assume an explanatory contribution of stressful work to the association of altered HRV with poor CHD prognostic outcomes.

HRV is also recognized as a biological indicator for work-related stress (48). For instance, HRV can serve as a metric for evaluating psychological stress experienced by emergency physicians during their duties (49). Several studies documented associations of high work stress with reduced HRV (50–52). For instance, concerning the JS model, four studies showed reduced HRV (53–56), whereas two more investigations did not support this notion (57, 58). With regard to the ERI model, a couple of investigations supported a link with reduced HRV (50–52, 57, 59–61). These studies were conducted among healthy workers in various trades and professions. Yet, no study on work stress and HRV was reported in post-MI patients so far.

It is of interest to observe that work stress in terms of ERI, but not of JS, was predictive in the current study. A similar finding was apparent in two earlier investigations of Chinese participants (39, 62). One explanation points to the relevant role of status-related and financial rewards emphasized in the former model, that may have received a prominent role in the context of rapid and far-reaching changes of working and employment conditions in China (63, 64). Clearly, additional research is required to support this notion, and at a more general level, cultural differences in the comparability of findings relating these work stress models with health outcomes between Western societies and societies with an Asian sociocultural background need to be critically appraised (65).

This study has several strengths. First, this was the first longitudinal study to examine the associations of work stress with HRV in patients after the first ACS. As a particular advantage of this study design, HRV was assessed four times within 1 year. Second, both independent and dependent variables in our study were measured with standardized instruments, with established psychometric quality criteria. Third, several further psychosocial factors (including anxiety, depression, and job burnout) were considered as potential confounders of the reported associations, given their documented associations with CHD prognosis (26, 27, 66).

A number of limitations need to be addressed. First, the proportion of female participants was relatively low, accounting for only 15.6%. Since the subjects included in this study were all at a working age, were relatively young, and were suffering from ACS, fewer women met these inclusion criteria. Second, work stress was assessed at baseline only, and potential changes of work stress and employment status after ACS were not documented. As a result, we are not able to explore the interplay between work stress and HRV over time. Third, we did not collect data on the types of occupations of our subjects. Therefore, the role of occupation in explaining associations between work stress and HRV is not able to be carefully examined. Fourth, unmeasured factors may have affected our HRV measurements, such as the participant’s physical condition, exercise regime, sleep, and breathing, thus potentially limiting the significance of reported findings. The results of this study have clinical implications for secondary prevention of coronary heart disease, particularly in view of the fact that the occurrence of this disease among younger age working populations was growing in recent past (67). To date, specific tailored measures of improving the quality of working and employment conditions among people with CHD returning to work are warranted. These measures include organizational changes instructed by the work stress models mentioned, suggesting the reduction of workload and the strengthening of control and reward at work (68). In addition, programs of medical rehabilitation among cardiac patients are now available that improve health outcomes, such as evidence-based cognitive behavioral therapy and stress management (69).

## 5 Conclusion

Our study found that work stress, as characterized by ERI, predicts a reduction in HRV over a one-year follow-up period after ACS onset. This underscores the significant potential role of work stress in the secondary prevention of ACS, as well as other types of CVD. Despite these promising results, it’s crucial to note that further studies are needed to confirm these findings and explore alternative mechanisms. Importantly, our study adds a valuable piece of evidence to understand the role of work stress in CVD secondary prevention, opening up new potential avenues for intervention, such as workplace-oriented and multidisciplinary programs (including cognitive behavioral therapy and coaching) which might be effective to job retention and disease management (17).

## Data availability statement

The original contributions presented in the study are included in the article/Supplementary material, further inquiries can be directed to the corresponding authors.

## Ethics statement

The studies involving humans were approved by The Ethics Committee of Kunming Medical University (Kunming, China). The studies were conducted in accordance with the local legislation and institutional requirements. The participants provided their written informed consent to participate in this study.

## Author contributions

ZH: Data curation, Funding acquisition, Investigation, Writing – original draft. XC: Investigation, Writing – original draft. PJ: Data curation, Investigation, Writing – original draft. BZ: Data curation, Investigation, Writing – original draft. YS: Data curation, Formal analysis, Validation, Writing – original draft. JS: Writing – review & editing. JL: Conceptualization, Methodology, Writing – review & editing. MZ: Conceptualization, Funding acquisition, Methodology, Project administration, Supervision, Writing – review & editing.
