# Supplementary material for: Work stress and changes in heart rate variability among employees after first acute coronary syndrome: a hospital-based longitudinal cohort study
Source: Front Public Health. 2024 Mar 26;12:1336065. doi: 10.3389/fpubh.2024.1336065 (PMC11005455; doi:10.3389/fpubh.2024.1336065)
Supplement: Supplementary file 1 [file Table_1.DOCX]

**Supplemental Table 1.** The raw HRV measures over 12 months after ACS [M (Q1, Q3)]

| HRV parameters | Baseline | 1 month | 6 months | 12 months |
| --- | --- | --- | --- | --- |
| Time domain HRV |  |  |  |  |
| SDNN (ms) | 122.39 (96.09, 161.30) | 150.57 (120.64, 207.43) | 168.29 (126.82, 238.07) | 175.12 (132.45, 272.15) |
| RMSSD (ms) | 58.84 (32.52, 136.59) | 95.92 (47.13, 189.87) | 100.93 (60.65, 192.89) | 113.87 (63.49, 209.57) |
| Frequency domain HRV |  |  |  |  |
| TP (ms^2^) | 7856.00  (3714.50, 19535.72) | 13167.00  (7186.79, 46166.05) | 18163.00  (8345.56, 58763.21) | 20587.65  (10342.85, 61236.36) |
| HF (ms^2^) | 1702.75  (475.00, 7555.27) | 4404.80  (1108.00, 12581.72) | 5583.75  (1345.43, 15685.23) | 5874.32  (1398.20, 17342.35) |
| LF (ms^2^) | 1466.00  (555.00, 4494.00) | 2384.00  (1018.00, 6974.39) | 2648.23  (1129.01, 7835.60) | 2978.68  (1238.97, 9423.42) |
| VLF (ms^2^) | 2018.28  (1312.91, 3987.00) | 3827.82  (2185.50, 5575.84) | 4036.29  (2320.22, 5892.90) | 4239.38  (2398.87, 6034.65) |
| ULF (ms^2^) | 2392.28  (1525.38, 5324.00) | 4722.06  (2722.00, 9520.00) | 4867.76  (2930.15, 10342.00) | 50435.20  (3129.01,11325.25) |

M: median; Q1: 25% quantile; Q3: 75% 25% quantile; HRV: heart rate variability; SDNN: standard deviation of NN intervals; RMSSD: root mean square of successive differences; TP: total power; HF: high frequency; LF: low frequency; VLF: very low frequency; ULF: ultra-low frequency.
